# Dynamic architecture of the *Escherichia coli* structural maintenance of chromosomes (SMC) complex, MukBEF

**DOI:** 10.1093/nar/gkz696

**Published:** 2019-08-10

**Authors:** Karthik V Rajasekar, Rachel Baker, Gemma L M Fisher, Jani R Bolla, Jarno Mäkelä, Minzhe Tang, Katarzyna Zawadzka, Oliwia Koczy, Florence Wagner, Carol V Robinson, Lidia K Arciszewska, David J Sherratt

**Affiliations:** 1 Department of Biochemistry, University of Oxford, South Parks Road, Oxford OX1 3QU, UK; 2 Chemistry Research Laboratory, Department of Chemistry, University of Oxford, 12 Mansfield Road, Oxford OX1 3TA, UK

## Abstract

Ubiquitous Structural Maintenance of Chromosomes (SMC) complexes use a proteinaceous ring-shaped architecture to organize and individualize chromosomes, thereby facilitating chromosome segregation. They utilize cycles of adenosine triphosphate (ATP) binding and hydrolysis to transport themselves rapidly with respect to DNA, a process requiring protein conformational changes and multiple DNA contact sites. By analysing changes in the architecture and stoichiometry of the *Escherichia coli* SMC complex, MukBEF, as a function of nucleotide binding to MukB and subsequent ATP hydrolysis, we demonstrate directly the formation of dimer of MukBEF dimer complexes, dependent on dimeric MukF kleisin. Using truncated and full length MukB, in combination with MukEF, we show that engagement of the MukB ATPase heads on nucleotide binding directs the formation of dimers of heads-engaged dimer complexes. Complex formation requires functional interactions between the C- and N-terminal domains of MukF with the MukB head and neck, respectively, and MukE, which organizes the complexes by stabilizing binding of MukB heads to MukF. In the absence of head engagement, a MukF dimer bound by MukE forms complexes containing only a dimer of MukB. Finally, we demonstrate that cells expressing MukBEF complexes in which MukF is monomeric are Muk^−^, with the complexes failing to associate with chromosomes.

## INTRODUCTION

**Figure 1.**
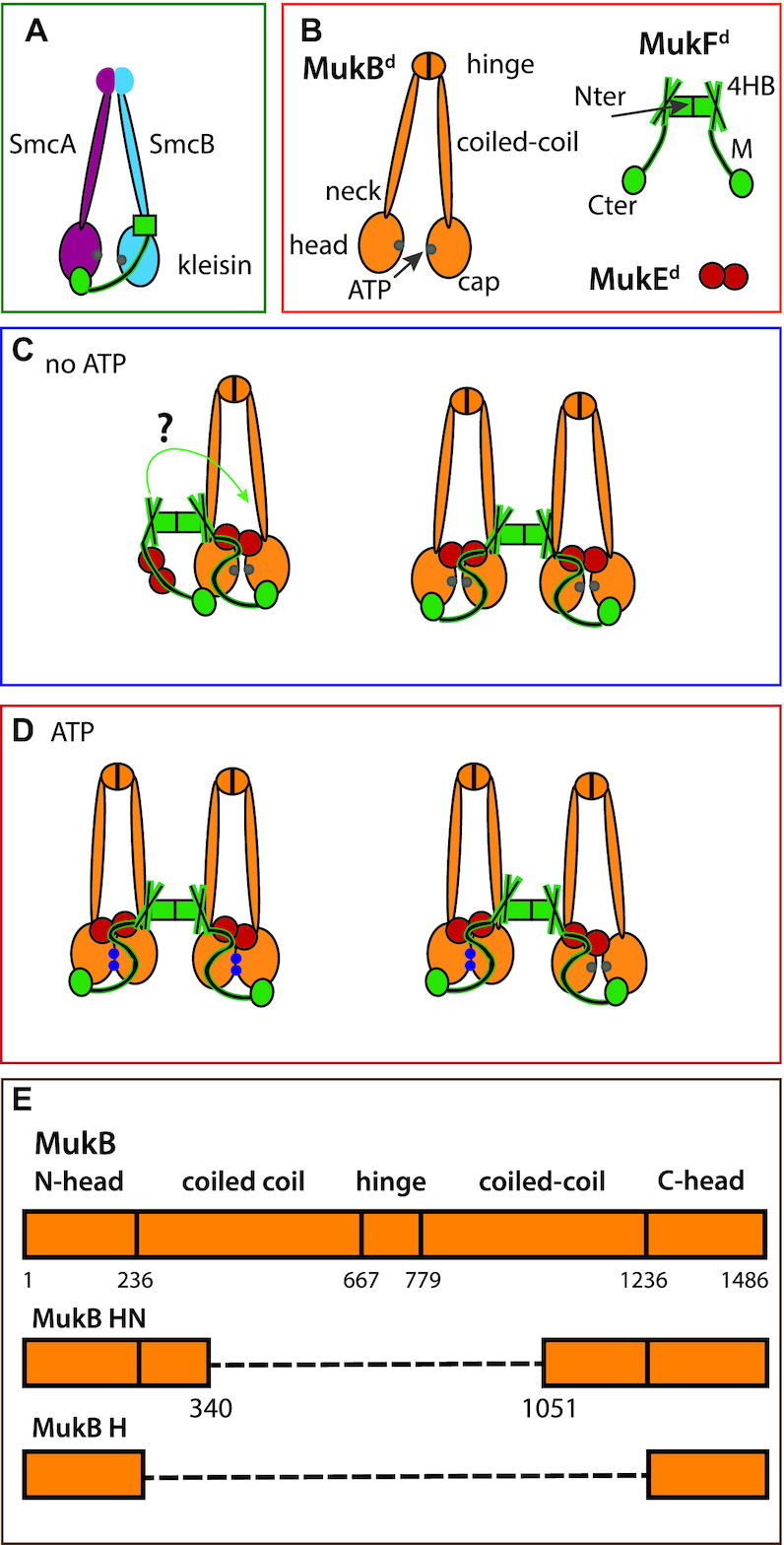
Schematics showing conserved SMC architectures and possible MukBEF stoichiometries and architectures. (**A**) Generic SMC complex architecture showing the tripartite proteinaceous ring formed by the kleisin and SMC proteins. (**B**) The components of the MukBEF complex. (**C** and **D**) Possible MukBEF architectures, without (C) and with (D) ATP. In D, the right panel shows a possible complex after ATP is hydrolysed in one of the dimers of a dimer of dimer complex. When ATP-dependent heads engagement occurs, the MukF middle region blocks the binding of a second MukF C-terminal domain to the second MukB head of a MukB dimer (27). When heads are unengaged, each MukB head can bind a MukF C-terminal domain, potentially leading to ‘daisy chain’ forms of a higher complexity than dimers and dimers of dimers (not shown). ATP-bound ATPase active sites denoted as blue dots on the heads and ADP-bound or nucleotide-unbound as grey dots. (**E**) Schematic showing MukB and its truncated variants head neck (HN) and head (H); a dashed line indicates a linker connecting N-and C-terminal head domains.

Structural Maintenance of Chromosomes (SMC) complexes, which are present in all domains of life, share a distinctive architecture in which a tripartite proteinaceous ring is formed by a dimer of two SMC molecules and a kleisin that connects the two SMC adenosine triphosphatase (ATPase) heads. The connection is provided through interactions of a kleisin C-terminal domain with the cap of an SMC head and the kleisin N-terminal region with a coiled-coiled ‘neck’ adjacent to the head of the partner SMC molecule (Figure 1A, 1–4). Emerging evidence supports the view that SMC complexes are mechanochemical motors that use cycles of ATP binding and hydrolysis to transport themselves rapidly with respect to DNA, extruding DNA loops during this transport (5). Such activities have important roles in chromosome organization-individualization and segregation, as well as other aspects of DNA management (6–9).

Although the *Escherichia coli* SMC complex, MukBEF, shares many aspects of the distinctive SMC complex architecture, its kleisin, MukF, is dimeric, which could potentially facilitate the formation and action of higher order complexes (Figure 1B–D; 6,10,11). MukBEF homologues are only found in a fraction of γ-proteobacteria, where they have co-evolved with a group of other proteins, including MatP, Dam and SeqA (12). MukBEF also coordinates the localization and action of TopoIV (13,14) with MatP-*matS* regulating the distribution and activity of both MukBEF and TopoIV in cells (15).

In *E. coli* cells, ∼200 dimeric MukBEF complexes (or their multimeric equivalent) are present, with ∼50% of these being tightly associated with chromosomal DNA, of which 30–50% form clusters in which the functional units are dimers of MukBEF dimers or multiples thereof (11). Clusters of wild-type MukBEF complexes are positioned at mid-cell in newborn cells and the cell quarter positions thereafter by a ‘phase-locked Turing pattern’ (16). These clusters position the chromosome replication origin region (*ori*) (15,17), thereby facilitating chromosome organization and segregation. ATP binding and MukB head engagement are essential for the formation of MukBEF clusters, as they are present in wild-type and in hydrolysis-deficient mutant (MukB^EQ^) cells, but not in cells impaired in nucleotide binding or in head engagement (11).

To help understand how MukBEF performs its functions in chromosome management, we analysed changes in the architecture and stoichiometry of MukBEF complexes *in vitro* as a function of nucleotide binding and hydrolysis. Using a combination of biochemical and biophysical approaches on truncated and then full-length MukBEF complexes, we have demonstrated that dimers of head-engaged MukBEF dimers form *in vitro* when bound to AMPPNP, a non-hydrolysable analogue of ATP, or to ATP when hydrolysis is impaired. We have shown the role of MukE in the formation of these dimers of dimers and present insight into the architectures of complexes with engaged and unengaged heads. Furthermore, we have demonstrated that cells expressing MukBEF complexes in which MukF is monomeric have a Muk^−^ phenotype, with the complexes failing to associate stably with chromosomes, thereby underlining the importance of MukF dimerization in MukBEF function.

## MATERIALS AND METHODS

### Protein purification

The proteins were expressed and purified as described (4), except MgCl_2_ (1 mM) was present during some purifications. MgCl_2_ is not necessary during the purification procedure and the proteins obtained ±MgCl_2_ have indistinguishable properties in our assays.

### Size exclusion chromatography and multi-angle light scattering (SEC-MALS)

Proteins were mixed at the indicated ratios and equilibrated in 50 mM HEPES pH 7.5, 100 mM NaCl, 1 mM MgCl_2_, 1 mM Dithiothreitol (DTT) and 10% glycerol (v/v) buffer supplemented with 1 mM adenosine diphosphate (ADP), ATP or AMPPNP for 3 h at room temperature. A total of 100 μl of these mixtures (containing 50–150 μg of total protein) were loaded onto either a Superose 6 HR10/30 column, or a Superdex 200 HR10/30 column (GE), equilibrated with the same buffer lacking glycerol and nucleotides. The separation was conducted at a flow rate of 0.5 ml/min. Presence of DTT or Tris(2-carboxyethyl)phosphine (TCEP) as reductants did not influence the results. Size exclusion chromatography and multi-angle light scattering (SEC-MALS) analysis was performed at 22°C using a Shimadzu (Kyoto, Japan) chromatography system, connected in-line to a Heleos8+ multi angle light scattering detector and an Optilab T-rEX refractive index (RI) detector (Wyatt Technologies, Goleta, CA, USA). Results were processed and analysed using ASTRA 6 (Wyatt Technologies).

### Native and SDS polyacrylamide gel electrophoresis (PAGE)

Sodium dodecyl sulphate-polyacrylamide gel electrophoresis (SDS-PAGE) were prepared as described (4). 6% native polyacrylamide gels were poured in 125 mM Tris-Cl buffer pH 8.8. Gels were run using Tris-Glycine running buffer (25 mM Tris–Cl, 192 mM glycine). Purified proteins were mixed at respective ratios in 50 mM HEPES pH 7.5, 100 mM NaCl, 1 mM MgCl_2_ and 1 mM DTT along with the respective nucleotide (1 mM ADP/ATP or AMPPNP) and equilibrated for 3 h at room temperature. Samples were mixed with glycerol to 20% (v/v) before loading. Gels were run at 35 mA for 30–35 min and stained using Instant Blue. For 2D PAGE analysis, native gel complexes were excised from the gel, and the gel slices were incubated with buffer containing 0.1% SDS and placed in the wells of 4–20% gradient Tris-glycine gel prior to electrophoresis.

### Isothermal calorimetry (ITC)

Reaction samples containing MukE (400 μM) and MukF (20 μM) were equilibrated in 50 mM HEPES pH 7.5, 100 mM NaCl, 1 mM MgCl_2_ and 1 mM DTT. Binding was assayed in a Malvern PEAQ ITC instrument at 25°C. Averages and standard deviations of the obtained parameters are reported from triplicate experiments. Data were analysed using the manufacturer's software assuming a single binding site model.

### Native-state ESI-MS spectrometry

Prior to MS analysis, protein samples were buffer-exchanged into 200 mM ammonium acetate pH 8.0, using a Biospin-6 (BioRad) column and introduced directly into the mass spectrometer using gold-coated capillary needles (prepared in-house; 18). Data were collected on a modified QExactive hybrid quadrupole-Orbitrap mass spectrometer (Thermo-Fisher Scientific) optimized for analysis of high-mass complexes, using methods previously described (19). The instrument parameters were as follows: capillary voltage 1.2 kV, S-lens RF 100%, quadrupole selection from 2000 to 20 000 m/z range, collisional activation in the HCD cell 200 V, argon UHV pressure 1.12  ×  10−9 mbar, temperature 60°C, resolution of the instrument 17 000 at m/z  =  200 (a transient time of 64 ms) and ion transfer optics (injection flatapole, inter-flatapole lens, bent flatapole, transfer multipole: 8, 7, 6 and 4 V, respectively). The noise level was set at 3 rather than the default value of 4.64. No in-source dissociation was applied. Where required, baseline subtraction was performed to achieve a better-quality mass spectrum.

### Proteomic analysis

MukB protein was digested with trypsin overnight at 37°C as described (20). Peptides were separated by nano-flow reversed-phase liquid chromatography coupled to a Q Exactive Hybrid orbitrap mass spectrometer (Thermo Fisher Scientific). The peptides were trapped onto a C18 PepMap 100 pre-column (inner diameter 300 μm × 5 mm, 100 Å; Thermo Fisher Scientific) using solvent A (0.1% formic acid in water) and separated on a C18 PepMap RSLC column (2 μm, 100 Å; Thermo Fisher Scientific) using a linear gradient from 7 to 30% of solvent B (0.1% formic acid in acetonitrile) for 30 min, at a flow rate of 200 ml/min. The raw data were acquired on the mass spectrometer in a data-dependent mode. Typical mass spectrometric conditions were: spray voltage of 2.1 kV, capillary temperature of 320°C. MS spectra were acquired in the orbitrap (m/z 350−2000) with a resolution of 70 000 and an automatic gain control (AGC) target at 3 × 10e6 with maximum injection time of 50 ms. After the MS scans, the 20 most intense ions were selected for HCD fragmentation at an AGC target of 50 000 with maximum injection time of 120 ms. Raw data files were processed for protein identification using MaxQuant, version 1.5.0.35 and searched against the UniProt database (taxonomy filter *E*. *coli*), precursor mass tolerance was set to 20 ppm and MS/MS tolerance to 0.05 Da. Peptides were defined to be tryptic with a maximum of two missed cleavage sites. Protein and peptide spectral match false discovery rate was set at 0.01.

### ATP hydrolysis assays

ATP hydrolysis was analysed in steady state reactions using an ENZCheck Phosphate Assay Kit (Life Technologies) as described previously (4), except the buffer contained 40 mM NaCl and 1.33 mM ATP.

### Functional analyses *in vivo*

The ability of a MukF monomer variant to complement the temperature-sensitive growth defect of a *ΔmukF* strain (AB233, AB1157 Δ*mukF mukBgfp*) in rich medium, was tested using basal expression from pET21, in the absence of Isopropyl beta-D-1thiogalactopyranoside (IPTG). Cells were transformed with pET21 carrying either *mukF* or *mukF*^M^ and allowed to recover for 8 h at a permissive (22°C) temperature, then 10^−2^ dilutions were spotted in duplicate onto LB plates containing carbenicillin (100 μg/ml) and incubated at either 22°C or 37°C.

### Epifluorescence Microscopy

Single colonies of cells were inoculated into M9 glucose (2%) and grown ON at 22°C. Cells were sub-cultured into the same medium and grown to A_600_ 0.1–0.2. For imaging, cells were spun and spotted onto an M9-glu 1% agarose pad on a slide. Microscope images were acquired on a Nikon Ti-E inverted microscope equipped with a perfect focus system, a 100 × NA 1.4 oil immersion objective (Nikon), an sCMOS camera (Hamamatsu Flash 4), a motorized stage (Nikon), an LED excitation source (Lumencor SpectraX) and a 30°C temperature chamber (Okolabs). Fluorescence images were collected with 200 ms exposure time using excitation from a LED source at 50% at 485 and 508 nm for GFP and mYpet, respectively. Phase contrast images were collected for cell segmentation. Images were acquired using NIS-Elements software (Nikon). Cell segmentation and spot detection from the fluorescence channel were performed using SuperSegger (21). Low quality spots were filtered out with a fixed threshold that was kept the same for all samples and the percentages of cells containing one or more spots were calculated using MATLAB (MathWorks).

## RESULTS

### MukF dimers direct formation of dimers of heads-engaged MukB dimers

To reveal the architectures and stoichiometries of MukBEF complexes experimentally, a truncated derivative of MukB, MukB_HN,_ (MukB Head-Neck, subsequently abbreviated as HN) containing the MukB ATPase head and ∼30% of the adjacent coiled-coil (Figure 1E), was used in initial biochemical analyses. This coiled-coil region contains the ‘neck’ to which the 4-helix bundle of MukF, adjacent to the N-terminal dimerisation domain, binds and activates MukB ATPase activity (Figure 1A and B; (4)). This strategy was chosen initially because of the technical challenges of incisive *in vitro* analysis of large ∼1 MDa full-length MukBEF complexes. A MukF dimer has four independent interfaces for binding MukB; the two MukF C-terminal domains and two N-terminal 4-helix bundles, which bind the MukB head and neck respectively (Figure 1B and D). Therefore, each MukF dimer could bind between two to four MukB molecules.

**Figure 2.**
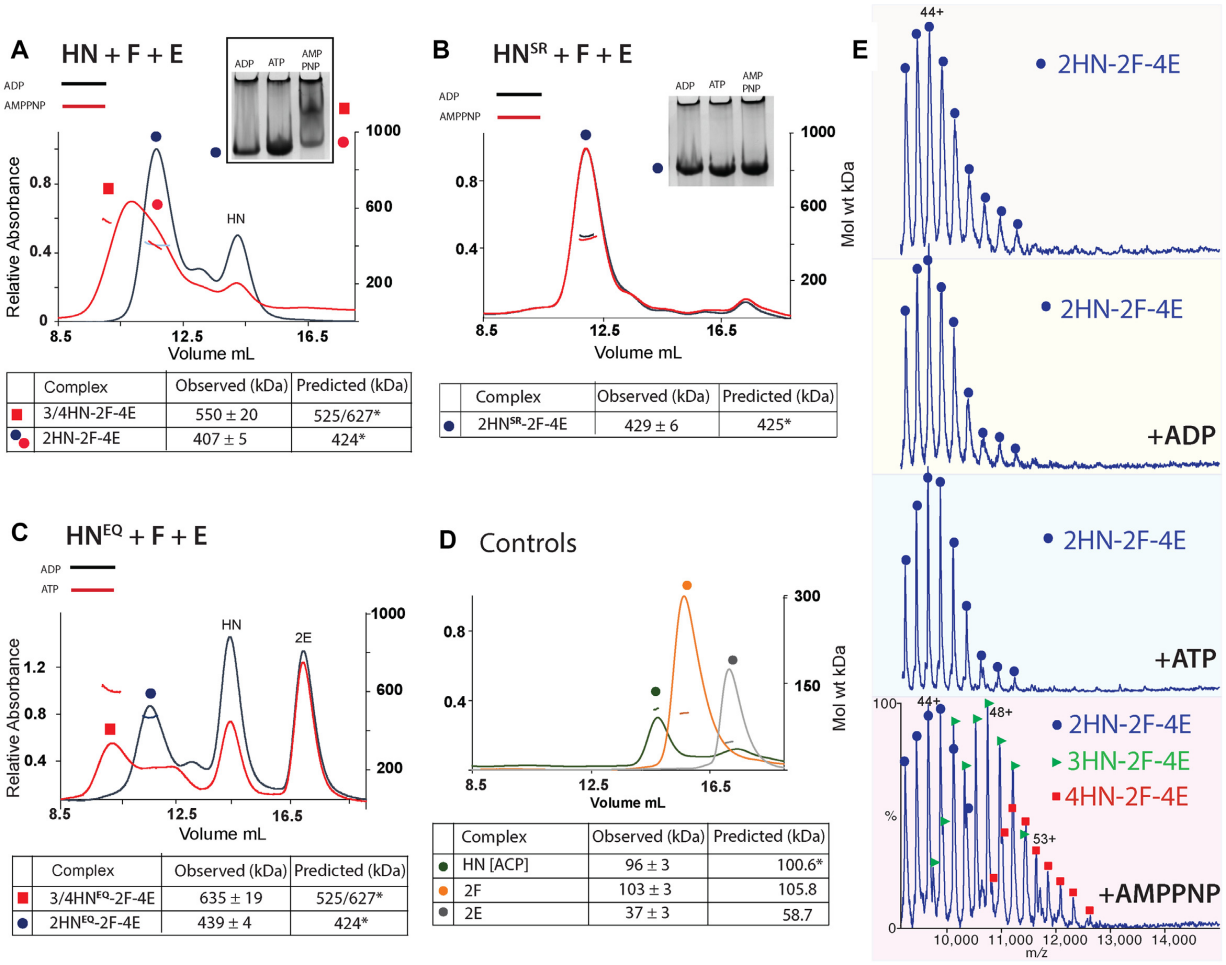
MukB head engagement is required for the formation of dimer of dimer MukBEF complexes. (**A**–**C**) Native PAGE (A and B) and SEC-MALS (A–C) analyses of the stoichiometry of HN/HN^SR^/HN^EQ^ complexes with MukFE in the presence and absence of ATP/AMPPNP. A total of 10 μM HN, 5 μM F and 10 μM E were incubated for 3 h at room temperature with ADP or AMPPNP/ATP (1 mM) prior to loading onto a 6% native gel, or a Superose 6 column. (**D**) SEC-MALS analysis of individual Muk proteins. The observed MukE mass of 37 kDa is consistent with previously published studies (30,31) and likely reflects MukE in an equilibrium mixture of monomers and dimers in solution. Predicted and observed masses of the complexes are tabulated below with the values and their uncertainties derived from a single representative SEC-MALS experiment. Predicted masses (kDa) of complexes containing ACP-4’phosphopantetheine bound to HN at stoichiometric levels (see below, Supplementary Figure S2) and bound AMPPNP when appropriate. Differences between the predicted and observed masses of the complexes are within 5%. (**E**) Native mass spectra of MukB HN complexes formed with MukFE in the absence of nucleotide or in the presence of ADP, ATP or AMPPNP. The complexes are indicated as follows: 2HN-2F-4E (blue dots), 3HN-2F-4E (green triangles) and 4HN-2F-4E (red squares). The observed and predicted masses (Da) of these complexes are tabulated in Supplementary Table S2.

SEC-MALS analysis revealed that HN formed complexes with MukEF, in the presence of AMPPNP, a non-hydrolyzable analogue of ATP (Figure 2A). The broad red peak appeared to be composed of two major components: material in the leading edge having a mass of 550 kDa (red square) and material in the lagging edge (red spot) with a mass of ∼404 kDa (Figure 2A and Supplementary Table S1). Material at the leading edge likely contained a mixture of 3/4HN-2F-4E complexes (red square), whilst material at the lagging edge was likely the 2HN–2F–4E complex (red spot). The 3/4HN–2F–4E complexes are expected to have one or two pairs of AMPPNP bound engaged heads, respectively, equivalent to a dimer of dimers MukBEF complex when MukB is a full-length wild-type dimer (Figure 1D). SEC-MALS of samples with ADP revealed just the presence of the ∼407 kDa complex, the mass of a 2HN–2F–4E complex (blue spot), which is equivalent to a dimeric MukBEF complex. Consistent with this interpretation, native gel electrophoresis demonstrated the AMPPNP-dependent formation of a slower moving complex (Figure 2A; upper panel; red square), along with faster running putative 2HN–2F–4E complexes formed in the presence of ADP (blue and red spots). 2D native-SDS-PAGE analysis confirmed the presence of the indicated proteins in the complexes, but was not sufficiently quantitative to confirm their stoichiometry (Supplementary Figure S1).

To assess the stoichiometry accurately, we conducted native mass spectrometry analysis of samples containing HN and MukEF in the presence of ATP/AMPPNP/ADP or in the absence of nucleotide (Figure 2E). Complexes with masses corresponding to 3HN/4HN-2F-4E were only observed in the presence of AMPPNP (Figure 2E and Supplementary Table S2). The native mass analysis, alongside subsequent proteomic analysis (data not shown), revealed that MukB HN, as well as full length MukB, was associated with ACP, Acyl Carrier Protein (likely bound to its prosthetic group, 4’phosphopantetheine) (22) at near stoichiometric levels (Supplementary Figure S2). The presence of ACP in purified MukB fractions has been reported previously (23–25). Any functional relevance to the association of ACP with MukB or HN remains to be determined. These results demonstrate AMPPNP-dependent formation of dimers of engaged-head dimer complexes. Incubation of samples with ATP resulted in the same mass spectrometry and electrophoretic profiles as incubation with ADP (Figure 2A and E), presumably because the ATP in any given complex was hydrolyzed before analysis under the conditions used. A HN^SR^ derivative that is deficient in head engagement (11,26), (Supplementary Table S3) failed to give the equivalent dimer of dimer complexes on addition of AMPPNP in SEC-MALS and native PAGE (Figure 2B), thereby providing further support for the interpretation that MukB head engagement is required for the formation of dimer of dimer complexes. Analysis of HN^EQ^, which binds ATP but is impaired in its hydrolysis as a consequence of the Walker B motif mutation (11,26), (Supplementary Table S3), showed that it forms the equivalent of dimer of dimer complexes in the presence of MukEF and ATP (Figure 2C), supporting our interpretations. Control experiments showed that HN was monomeric because of the lack of a dimerization hinge, whilst MukF and MukE were dimeric, as expected (Figure 2D).

**Figure 3.**
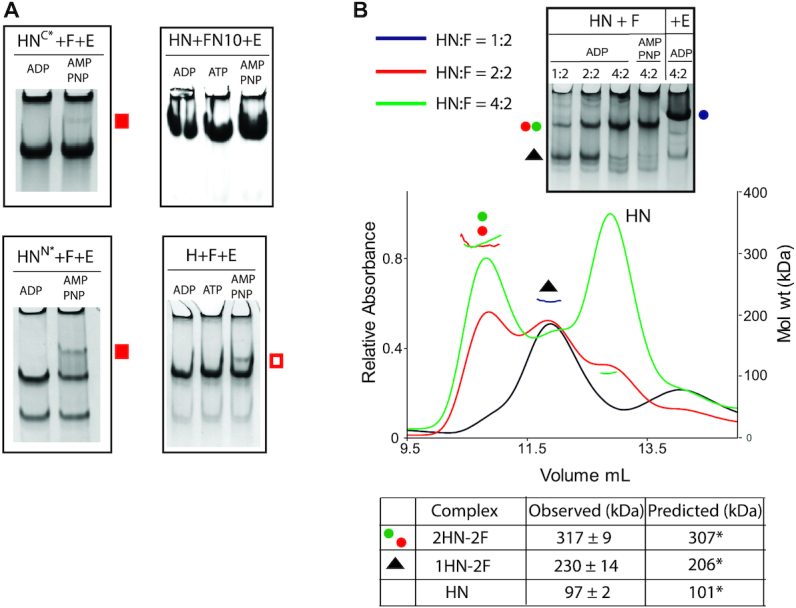
Dimer of dimer formation requires MukE, and HN interactions with both the MukF C-terminal domain and the MukF 4-helix bundle. (**A**) Native PAGE of complexes generated with HN and F variants deficient in binding across one (HN^C*^, FN10), or the other (HN^N*^and H), HN-F interface. The position of low levels of dimer of dimer complexes is indicated with filled red squares (HN), or an unfilled red square (H). HN^C*^ carries the following aa residues alterations: F1453S, H1458A, R1465A. (**B**) SEC-MALS analyses of HN-F complexes at different HN:F ratios; 2.5/5/10 μM HN was mixed with 5 μM F prior to separation through a Superdex 200 column. Predicted and observed masses of the complexes are tabulated below. An 11% difference between the predicted and observed mass of 1HN-2F reflects a presence of some 2HN-2F in the peak (black trace). The same mixtures incubated with ADP along with samples containing 10 μM HN+5 μM F and either AMPPNP or 10 μM E were analysed on 6% native gels.

To test whether interactions of HN with both the MukF C-terminal domain and the 4-helix bundle adjacent to the MukF N-terminal domain are necessary to form dimer of dimer complexes, MukEF were incubated with HN^C*^, which is predicted to be deficient in its interaction with the MukF C-terminal domain as it carries amino acid substitutions at the binding interface (27), (Figure 3A and Supplementary Table S3). Only a trace of 4HN^C*^–2F–4E complexes was observed in the presence of AMPPNP (filled red square), thereby demonstrating that interaction of the MukF C-terminal domain with the MukB cap is essential for the formation of dimers of dimers. Supporting this conclusion, incubation of HN with FN10, lacking the MukF C-terminal domain (4) and MukE (Figure 3A), generated no dimer of dimer complexes in the presence of AMPPNP. Equally, interaction of MukEF with the MukB head (H), lacking the neck, or a mutant in the neck that impairs interaction with the MukF 4-helix bundle, HN^N*^, (4) (Supplementary Table S3), also led to a much reduced level of dimer of dimer complexes in the presence of AMPPNP (unfilled and filled red squares, respectively). We conclude that interaction of both the MukF 4-helix bundle with the MukB neck and the interaction of the MukF C-terminal domain with the cap on the MukB head are crucial for efficient dimer of dimer formation, with the MukF C-terminal interaction with the head having a more important role than the interaction of the MukF 4-helix bundle with the neck.

We then addressed whether MukE is required to form AMPPNP-dependent heads-engaged dimer of dimer complexes and whether the absence of MukE could influence the stoichiometry of complexes. Incubation of HN with MukF dimers at varying molar ratios produced complexes with a molecular mass of ∼317 kDa in the presence of ADP, close to the mass expected of 2HN–2F complexes (Figure 3B; red and green spots). At a ratio of HN: MukF of 0.5, most material eluted within a peak with a mass of 230 kDa, as predicted for HN–2F complexes (black triangle). In native PAGE, the same titrations in the presence of ADP showed the formation of a slower migrating complex (red and green dots), which increased in abundance as the relative HN concentration increased; we interpret this complex as 2HN–2F. Because replacement of ADP by AMPPNP made little difference to the complexes’ mobility, we conclude that MukE is required to form dimer of dimer complexes. The native gel also shows how MukE influences the mobility of HN–MukF complexes (compare blue spot with red/green spots).

Consistent with previous results (28,29) MukE bound tightly to a MukF dimer in SEC-MALS and native PAGE analyses (Supplementary Figure S3A). Isothermal calorimetry (ITC) assays revealed a dimeric MukF binding to two MukE dimers with a *K*_d_ of 6.97 ± 2.6 nM (Supplementary Figure S3B). We failed to detect interaction between MukE and HN in the absence of MukF in our biochemical analyses (Supplementary Figure S4), despite crystal structures showing interaction surfaces between MukE and MukB heads (27).

Although the assays described here are suitable for detecting the equivalent of MukBEF dimers of dimers, as judged by dimeric MukF molecules capturing four molecules of HN, complexes containing a dimeric MukF molecule bound by two HN molecules could correspond to either dimeric full-length MukBEF complexes, or to dimers of MukBEF dimers. Subsequent experiments were designed to help resolve this ambiguity.

### Further characterization of MukBEF architecture and stoichiometry

**Figure 4.**
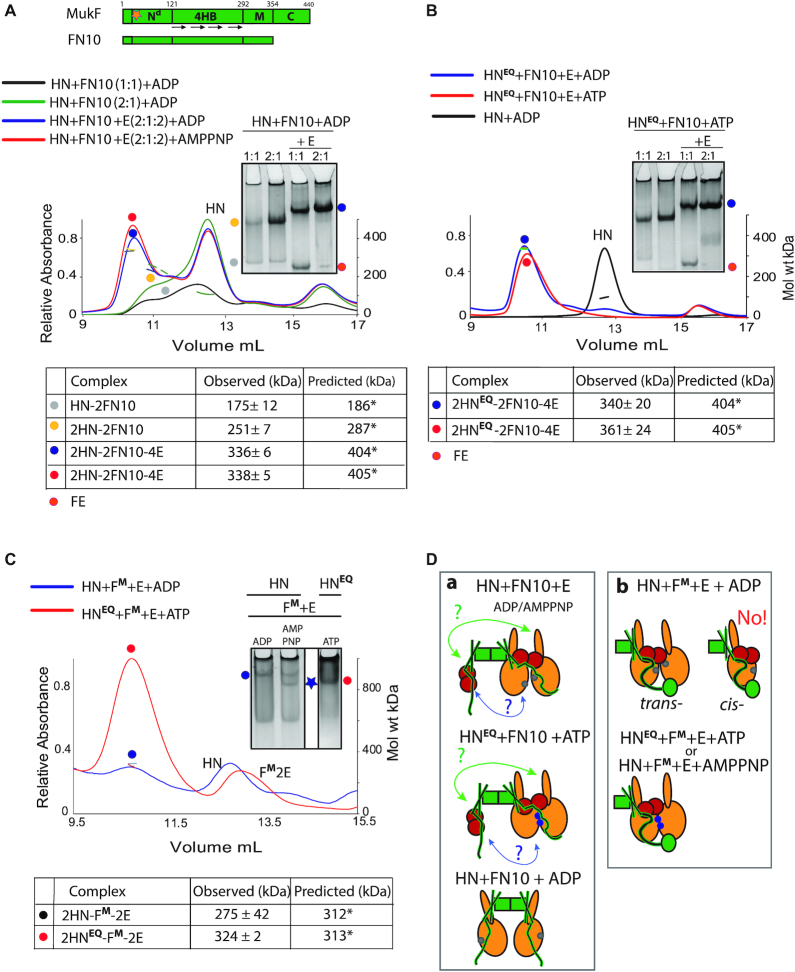
Architecture of MukBEF complexes. (**A**) Top, schematic of MukF and its truncated derivative FN10; N**d**, N-terminal dimerisation domain; 4HB, 4-helix bundle; M, middle region; and C, C-terminal domain, which is deleted in FN10. Orange star indicates position of dimerisation interface, which has been altered in MukF**^M;^** also see (Figure 6). (A and**B**) SEC-MALS and native PAGE analyses of HN and HN^EQ^ complexes generated with FN10 dimers. For SEC-MALS, samples at the indicated protein ratios were incubated with ADP, AMPPNP or ATP before separation through a Superose 200 column. FN10 was at 5 μM and E, when present, was 10 μM. Predicted and observed masses of complexes are tabulated below traces. Native gel samples were incubated with ADP (A) or ATP (B) prior to loading onto a gel. (**C**) SEC-MALS and native PAGE analyses of HN and HN^EQ^ complexes generated with F^M^-E in the presence of ADP, AMPPNP or ATP as indicated. The proteins were at concentrations of 10 μM HN/HN^EQ^, 5 μM F^M^ and 10 μM E. (A–C) Significant (<16%) differences in observed to predicted masses of the complexes in some experiments are due to incomplete resolution of the complexes from unbound HN. (**D**) Schematics of the proposed architectures with FN10 (panel a) and MukF^M^ (panel b). The green arrows (a) indicate a possible interaction between the FN10 4HB and the neck of the distal HN molecule. A second potential interaction between the ‘free’ FN10 middle region and its bound MukE to the proximal HN molecule is indicated by blue arrows. The bottom cartoon in (a) shows two HN molecules binding a FN10 dimer through interactions with the 4HBs. ATP-bound ATPase active sites denoted as blue dots on the heads and ADP-bound or nucleotide-unbound as grey dots.

To further characterize the dynamics of the MukBEF architecture, we analysed the interaction of HN with two different MukF derivatives. Dimeric FN10, lacking the MukF C-terminal domain (Figure 4A, top), interacts normally with MukE (4). Incubation of FN10 with HN produced complexes of masses expected for FN10 dimers with one or two bound HN molecules, 175 and 251 kDa, respectively (Figure 4A, yellow and grey spots). The equivalent complexes (yellow and grey spots) were inferred from native gel electrophoresis. The proportion of complexes with two HN molecules increased as the relative concentration of added HN increased in both SEC-MALS and native gel electrophoresis. We conclude that in the absence of MukE, the two 4-helix bundles of a FN10 dimer can each bind one HN neck (Figure 4D; panel a, bottom).

Although, the presence of MukE did not alter the relative stoichiometry of FN10 with HN, it led to a higher proportion of complexes with two HN molecules bound (compare green and blue trace), indicating that MukE stabilizes and perhaps ‘re-conforms’ these complexes (Figure 4D; panel a top). The SEC-MALS traces and observed masses of 2HN-2FN10–4E complexes were similar in the presence of ADP and AMPPNP (compare red and blue traces) suggesting an inability of this complex to support dimer of dimers formation, consistent with the earlier conclusion that interactions of the MukF C-terminal domain with the HN cap are required. Equivalent SEC-MALS and native PAGE profiles were observed when HN^EQ^, in the presence of ATP, was used rather than HN (Figure 4B). Since HN^EQ^ is predominantly dimeric upon incubation with ATP (Supplementary Figure S5), we conclude that only one pair of heads-engaged HN molecules can bind to a FN10 dimer in the presence of MukE. This is either because the necks of the two HN molecules in these complexes occupy both 4-helix bundles (Figure 4D; panel a, green arrows), or because binding of a HN neck to one 4-helix bundle prevents the second 4-helix bundle in a dimer from interacting with another neck because of conformational changes/steric clashes. We do not know if a ‘free’ FN10 middle region with bound MukE can interact with the second HN molecule (Figure 4D; panel a, blue arrows).

**Figure 5.**
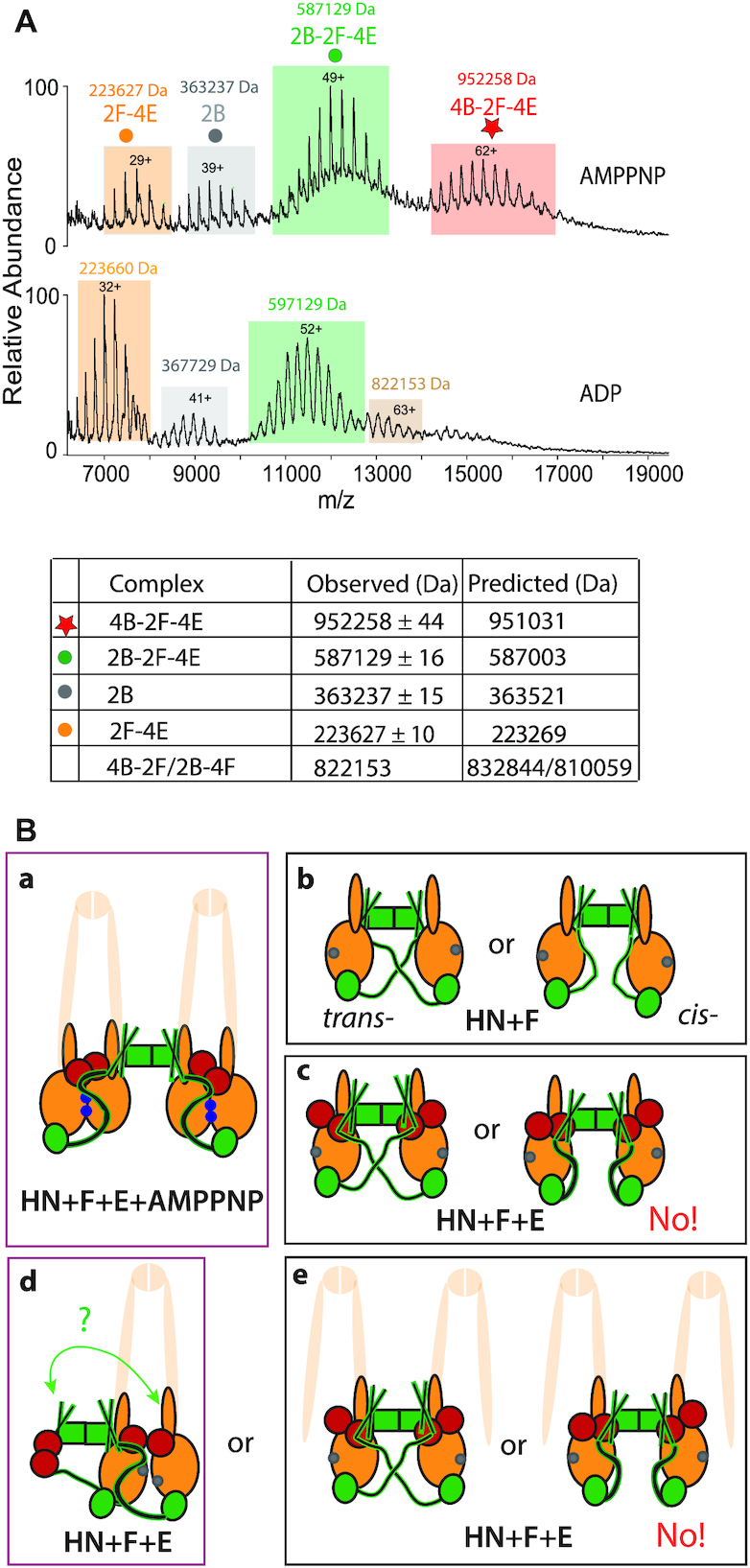
Dimers of dimers are formed with full length MukB dimers, MukEF and AMPPNP. (**A**) Native mass spectra of complexes formed with MukBEF in the presence of AMPPNP (top), or ADP (bottom). The predicted and observed masses are tabulated below the graphs. The proteins were at concentrations 5 μM B, 2.5 μM F and 5 μM E. The small population of complexes (mass 822153 Da, beige) observed in the ADP sample may reflect a presence of 4B-2F or 4B-2F-E2 complexes (predicted mass 832843.68 Da and 891578 Da, respectively). (**B**) Schematics of the architectures demonstrated or inferred from the biochemical analyses. The extrapolation from HN to full length MukB dimers is cartooned by showing the remainder of MukB as semi-transparent. Panel (**a**), MukBEF-AMPPNP- and head engagement-dependent dimer of dimers. (**b**) Alternative possible architectures of HN + MukF. (**c**) As (**b**) in the presence of MukE. The data provide evidence for the *trans*-configuration shown on the left. Panels **d** and **e**, show possible configuration of 2HN-2F-4E dimers in the presence of ADP/absence of head engagement. Note that the architectures in d and e-left are topologically identical if both necks engage with a 4-helix bundle (green arrow; see Figure 4D), although if part of a full-length MukB dimer as indicated, would be a dimeric MukBEF complex (d) or dimer of dimer complex (e). ATP-bound ATPase active sites denoted as blue dots on the heads and ADP-bound or nucleotide-unbound as grey dots.

Additionally, we used a monomeric MukF derivative (MukF^M^, see below) that is unable to form dimers because of amino acid substitutions at the dimerization interface, although it has an intact 4-helix bundle, MukE binding sites and C-terminal domain. MukF^M^ was incubated with either HN or HN^EQ^ and MukE (Figure 4C). SEC-MALS showed the formation of complexes containing two HN molecules bound to a single MukF monomer, with HN^EQ^-ATP giving a much higher fraction of such complexes (red spot), as compared to HN-ADP (blue spot) This result was corroborated by the native PAGE profiles. Therefore, in the presence of MukE, a single MukF monomer, with an intact C-terminal domain and 4-helix bundle, can bind two HN molecules, irrespective of whether they are in the heads-engaged (HN^EQ^-ATP or HN-AMPPNP), or unengaged state (HN-ADP). Unsurprisingly, the native gel shows a higher proportion of such complexes is present when the heads are engaged, with the complexes having a higher mobility, indicative of a more compact conformation (blue star). This result argues that a single HN molecule is unable to employ both its neck and cap with the 4-helix bundle and C-terminal domain of the same MukF polypeptide in the presence of MukE, which likely plays a role in directing this arrangement. Otherwise, MukF monomers bound by a single HN molecule would be the dominant species. Figure 4D summarizes the proposed architectures that are demonstrated when HN are complexed with FN10 in the absence and presence of MukE (panel a), or when HN was complexed with MukF monomer in the presence of MukE (panel b). The failure to observe the ‘*cis*-configuration’ in which the neck and head of a single MukB molecule bind both the 4-helix bundle and C-terminal domain of a single MukF monomer (panel b) is consistent with the *trans*-configuration being important in directing a tripartite proteinaceous SMC ring (Figure 1A, Figure 5B bc). The observation that a single heads-engaged HN^EQ^ dimer binds to both a FN10 dimer and a MukF monomer (Figure 4D; compare panels a and b) is consistent with the conclusion above that FN10 dimers bound by MukE can only bind two HN molecules irrespective of whether they are engaged or not (panel a).

### Full length MukB forms dimers of heads-engaged dimer complexes with MukEF and AMPPNP

To ascertain whether the AMPPNP- and MukE-dependent formation of the equivalent of dimer of dimer complexes, hitherto characterized with HN variant of MukB, could be observed with the intact MukB, we endeavoured to analyse complexes with full-length MukB. However, MukBEF complexes were not amenable to native PAGE analysis. Furthermore, available size exclusion chromatography columns have limited resolution in the size range expected for full-length MukBEF complexes (0.5–1 MDa). Nonetheless, a Superose 6 column, combined with MALS, granted sufficient resolution to observe changes in the mass of complexes in the presence of AMPPNP (Supplementary Figure S6, compare green and red traces). However, the mass measurements over-estimated the theoretical masses of all complexes: by up to 20% for smaller complexes (2B, 2B2F) and by above 20% for MukBEF complexes. For example, a mixture of MukB and MukF (B+F sample, blue trace) produced a dominant complex of 555 kDa, roughly corresponding to a complex formed by the interaction of a MukB dimer and a MukF dimer (2B-2F, blue dot; predicted mass, 452 kDa, 18% mass discrepancy). However, a minor faster-running, shallow and broad peak (blue triangle, mass 1252 kDa) was also apparent. This material was likely a mixture of 4B-2F or 2B-4F (predicted mass 797 and 810 kDa, respectively) and higher-order ‘daisy-chain’ species in which two or more MukF dimers have joined two or more MukB dimers.

When MukB was incubated with MukF, MukE and ADP, a complex of 720 kDa, consistent with the expected 2B-2F-4E (green dot; predicted mass 587 kDa, 29% discrepancy) was observed. Yet, when the same proteins were incubated with AMPPNP, a shoulder of faster eluting species with a leading edge mass of 1205 ± 35 kDa (red star) was detected (Supplementary Figure S6). We propose that the faster eluting complexes contain dimers of MukBEF dimer, 4B-2F-4E (predicted mass 951 kDa, 26% discrepancy), corresponding to MukE- and AMPPNP-dependent dimers of heads-engaged dimers.

We then used native mass spectrometry to analyse intact MukBEF complexes (Figure 5A). When MukB, MukF and MukE were incubated with ADP, the resulting mass spectra revealed three common charge state distributions corresponding to MukB dimers (2B, dark blue dot), MukF dimers bound by two MukE dimers (2F-4E, orange dot) and MukB dimers in complex with MukF dimers bound by two MukE dimers (2B-2F-4E, green dot). When AMPPNP was present in the sample, we detected a charge state series for a higher mass species that corresponded to 4B-2F-4E—the proposed dimer of heads-engaged MukBEF dimers (red star). Note that in the sample with ADP, there was also a small population of complexes (beige shading) that had a mass (822,153 Da), consistent with a 4B-2F or 2B-4F complexes (832 844 or 810 0159 Da, respectively; Figure 5A).

### *E. coli* cells expressing monomeric MukF are impaired in MukBEF function

**Figure 6.**
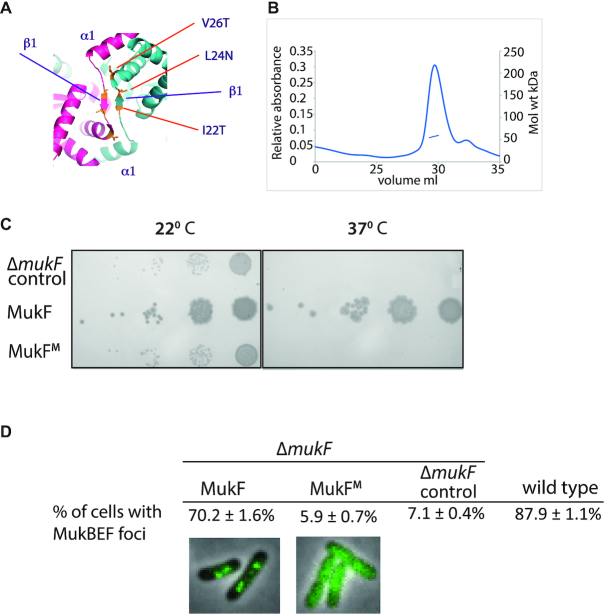
*Escherichia coli* cells expressing monomeric MukF have a Muk^−^ phenotype and fail to form MukBEF clusters on the chromosome. (**A**) Dimerisation interface of MukF showing the three mutated residues, I22T, L24N, V26T. (**B**) SEC-MALS of MukF monomers. The observed mass of 57.3 kDa corresponded to theoretical mass, of 52.9 kDa, of the monomeric variant. A small shoulder in the elution profile reflects the presence of the protein proteolytic cleavage product. (**C**) Temperature-sensitivity of growth in rich medium assay. 10^2^-fold serial dilutions of Δ*mukF* cells containing plasmid pET21 expressing basal levels of wild-type MukF or the MukF monomer (MukF^M^) are shown, alongside a control in which cells contain the plasmid vector alone. (**D**) Δ*mukF* cells expressing MukF monomers (MukF^M^) fail to form MukBEF foci. The analysis of foci formation was performed in Δ*mukF* strain carrying C-terminal *mukB-gfp* fusion; MukF and MukF^M^ were expressed from pET21 as in (C). For comparison, foci formation in a strain carrying the intact endogenous chromosomal copy of *mukF* was monitored using MukB-mYpet expression (SN192), (15).

To ascertain whether formation of dimers of dimers, directed by MukF dimers, is essential for MukBEF function, we used the MukF structure (27) to construct a variant, MukF^M^, that was predicted to be deficient in dimerization (Figure 6A). Purified MukF^M^ was monomeric in solution (Figure 6B) and it was biochemically active as judged by its ability to form complexes with MukE dimers and to stimulate the ATPase activity of MukB (Supplementary Figure S7 and Table S4).

We then assessed if monomeric MukF could support the function of MukBEF *in vivo*. The phenotype of cells expressing MukF^M^, using basal expression from plasmid pET21, in the presence of endogenous MukBE, was tested for temperature-sensitive growth on rich medium. The MukF^M^ expressing cells were as temperature-sensitive as a control Muk^−^ derivative, showing that MukF monomerization leads to the impairment of MukBEF function *in vivo* (Figure 6C). Moreover, the epifluorescence microscopy revealed that cells expressing monomeric MukF^M^ lacked MukBEF foci, demonstrating that MukBF^M^E complexes cannot stably interact with chromosomal DNA. We conclude that MukF dimerization is essential for *in vivo* MukBEF function.

## DISCUSSION

We have demonstrated the formation of MukBEF complexes having an architecture in which two MukBEF dimers are joined by a MukF dimer, forming dimer of dimer complexes. Furthermore, we show that MukF dimerization is essential for the MukBEF function *in vivo*, which is consistent with the observation from quantitative imaging that MukBEF dimers of dimers, or multiples thereof, are the functional unit *in vivo* (11). Analysis of the interactions of a truncated MukB derivative (HN), containing only the ATPase head and 30% of the adjacent coiled-coil, has demonstrated that the formation of these complexes is dependent on AMPPNP/ATP, MukE and MukB head engagement (Figure 5B, panel a). Likewise, we have provided evidence that dimer of dimer complexes are also generated *in vitro* with intact MukB protein. Moreover, our analysis shows that HN-EF dimers of dimers are converted back to dimers upon ATP hydrolysis, suggesting that MukBEF complexes function utilising ATP hydrolysis to switch between these two conformations.

One role of MukE appears to be to organize and stabilize the MukBEF complexes into a more compact form that allows head engagement upon ATP binding. Furthermore, MukE binding to MukF may ensure that the 4-helix bundle and C-terminal domain of a given MukF monomer bind to separate MukB heads (*trans*-configuration), thereby directing the formation of a kleisin-SMC tripartite proteinaceous ring (Figure 1). With the HN substrates, in the absence of head-engagement, 2HN-2F-4E dimers were the predominant form, but we cannot ascertain whether these correspond to MukBEF dimers, or dimers of MukBEF dimers (or both) (Figure 5B; compare panels d and e). Nevertheless, the analysis of full length dimeric MukB in complexes with MukFE and AMPPNP or ADP by native mass spectrometry, and SEC-MALS demonstrated that dimers of dimers form in the presence of MukBEF and AMPPNP, and that MukBEF dimers are the major component in the presence of ADP.

How does the demonstration of a switch between the dimer of dimers state and the dimeric state by replacing ATP/AMPPNP by ADP in the HN model system relate to the *in vivo* behaviour of MukBEF complexes (Figure 5B, compare panel a with d)? Because the experiments reported here use endpoint measurements in which MukB complexes are saturated with the added nucleotide (other than when added ATP can be hydrolyzed), we are unable to assess a possible *in vivo* situation in which a dimer of dimers undergoes ATP hydrolysis in both dimers simultaneously, leading to a MukBEF dimer (Figure 1, panel c, left). Alternatively, if ATP hydrolysis occurs in only one of the dimers at a given time, the dimer of dimer stoichiometry would be retained (Figure 1, panel d, right). Future work needs to address if both of the ATPase active sites in a MukB dimer undergo catalysis synchronously, and if there is any coordination of ATPase activity between the two MukB dimers in a dimer of dimers.

Although all four interaction interfaces between MukB and MukF (MukB neck-MukF 4HB, and MukB cap-MukF C terminal domain) can be occupied, our analyses revealed that in the absence of MukE, only two HN molecules could be bound stably at any one time. Similarly, in the presence of MukE and absence of head engagement only two HN molecules were stably bound, presumably in one of the configurations shown in Figures 4D, panel a and 5B, panel d. At present, we cannot distinguish the alternative models that can explain why only one pair of heads-engaged HN molecules can bind to a MukFE dimer. A model of MukF 4-helix bundles bound by MukB (4), based on available structural information, including a ‘symmetrical juxtaposed heads’ complex with two bound MukF C-terminal domains (27), indicates that a structural constraint could prevent the interactions shown by green and blue arrows in Figure 4D, panel a, and the equivalent interactions in full length MukF dimers. If the modelling is misleading, interactions between both HN necks and the two 4-helix bundles could occur (Figure 5B, panel d, green arrows and panel e, left). Note that if the two necks in Figure 5B, panel e, left were part of the same MukB dimer, then this architecture is essentially the same as shown in panel d, if the interaction indicated by the green arrow occurs.

The demonstration that MukF dimers can direct the formation of dimers of heads-engaged MukB dimers in the presence of MukE and ATP/AMPPNP, using both truncated MukB derivatives and the wild-type MukB, provides strong biochemical support for our inference of such complexes in active MukBEF clusters associated with *E. coli* chromosomes *in vivo*, using quantitative imaging (11). It therefore seems likely that all those bacteria whose genomes encode MukBEF, rather than the typical and more widely distributed SMC-ScpAB, will use a dimeric MukF to direct the formation of dimers of MukBEF dimers. Indeed, organisms belonging to γ-Proteobacteria contain highly conserved elements that provide the dimerisation interface characterised here, which are missing in other bacterial species (Supplementary Figure S8). Following our observation of putative dimers of dimers *in vivo*, we proposed that such complexes could be important in the transport of MukBEF with respect to chromosomal DNA by using a ‘rock-climber’ mechanism, in which the increased number of DNA–protein contact points in a dimer of dimers facilitates the transport (11). As yet we have not succeeded in obtaining direct evidence for a putative transport mechanism. We also consider two other possibilities that are not necessarily exclusive to a role in DNA transport. First, that the role of dimer of dimer complexes is related to interaction of MukBEF with MatP-*matS* or with topoisomerase IV and the consequent biological outcomes (14,15). Second, that dimer of dimer complexes are important for the proposed locked-phase Turing patterning mechanism that places MukBEF clusters at mid-cell or the cell quarter positions and thereby correctly positions replication origins, thereby facilitating chromosome segregation (16). It seems possible that all MukBEF orthologues use such a patterning system, along with acting in DNA transport, and hence the restriction of kleisin dimerization to MukBEF orthologues may relate to some specific property of these orthologues, other than (or in addition to) the DNA transport mechanism itself.

## Supplementary Material

gkz696_Supplemental_File

## Notes

Present address: Karthik V. Rajasekar, Evotec (UK), Abingdon, OX14 4SA, UK.

Present address: Minzhe Tang, The Francis Crick Institute, 1 Midland Road, London, NW1 1AT, UK.

Present address: Katarzyna Zawadzka, MNM Diagnostics, Wielkopolska Center For Advanced Technologies, 61-614 Poznań, Poland.

Present address: Oliwia Koczy, Department of Microbiology, Jagiellonian University, 30–387 Kraków, Poland.
