# Supplementary material for: Dynamic architecture of the Escherichia coli structural maintenance of chromosomes (SMC) complex, MukBEF
Source: Nucleic Acids Res. 2019 Aug 10;47(18):9696–707. doi: 10.1093/nar/gkz696 (PMC6765140; doi:10.1093/nar/gkz696)
Supplement: gkz696_Supplemental_File [file gkz696_supplemental_file.pdf]

Supplementary figure S1

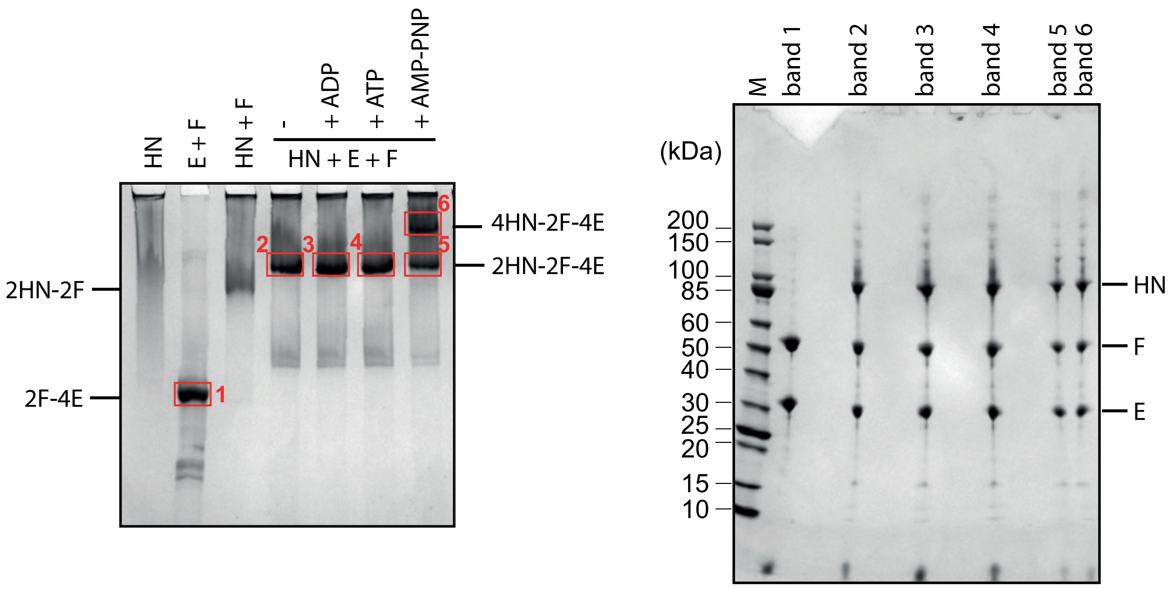

2D PAGE analysis of MukB HN complexes with MukFE . The protein samples containing MukBHN, MukF, MukE as in Figure 1A were resolved through 6% native PAGE. The indicated bands were cut out of the gel, soaked in SDS loading buffer and resolved in 4-20% gradient SDS PAGE.

Supplementary figure S2

Native MS

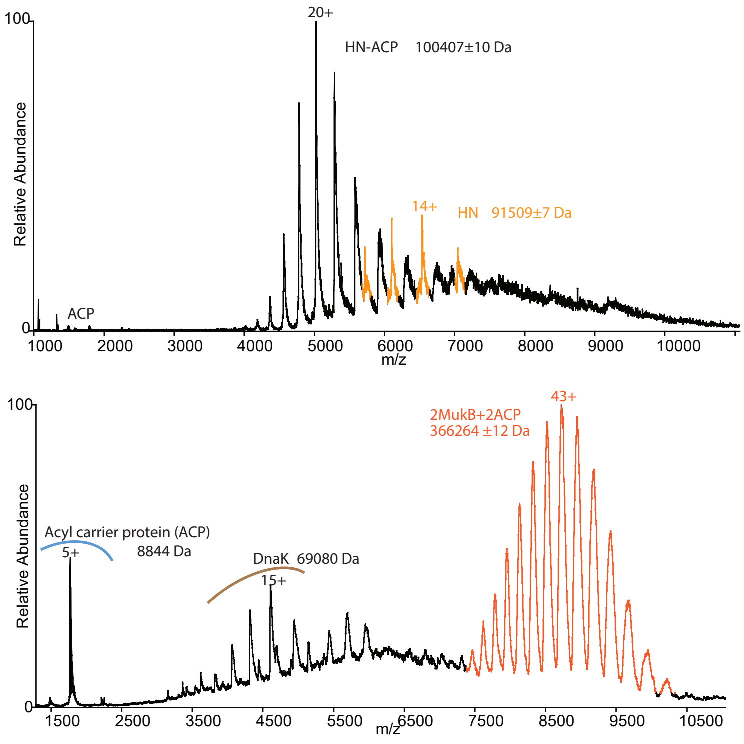

Mass spectrometry reveals stoichiometric binding of ACP-4’phosphopantetheine to MukB and MukBHN. **A** Native mass spectra of MukBHN and MukB. The predicted mass for MukBHN (His-tag) and MukBHN (his-tag)-ACP-4’phosphopantetheine are 91584.7 Da and 100224.22Da, and for 2MukB (His-tag) and 2MukB (His-tag) – 2ACP-4’phosphopantetheine are 345561.74Da and 362840.75Da, respectively.

Supplementary figure S3

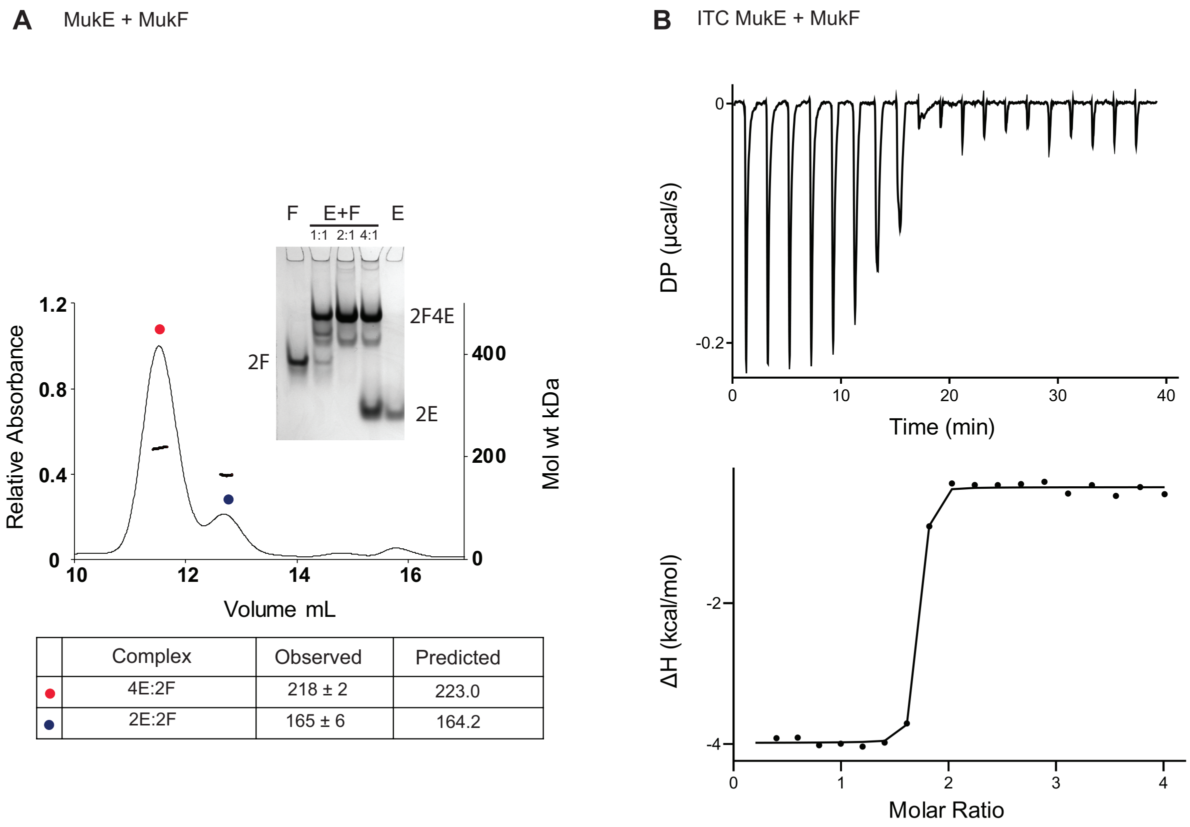

	Complex	Observed	Predicted
● (red)	4E:2F	218 ± 2	223.0
● (blue)	2E:2F	165 ± 6	164.2

Tight interaction between MukF and MukE. **A** SEC-MALS of MukE-MukF complexes on Superdex 200 column with 100 µL of 10 µM MukF and 20 µM MukE. Inset figure shows a 6 % native PAGE. Lanes from left to right are 5 µM MukF, 5 µM MukE + 5 µM MukF, 10 µM MukE + 5 µM MukF, 20 µM MukE + 5 µM MukF, 5 µM MukE. **B** ITC binding isotherm of 400 µM MukE titrated into 20 µM MukF at 25 °C in an ITC 200. Fitted parameters for a single binding site model from three independent measurements were N=1.87 ± 0.24, K_d=6.97 ± 2.6 nM, ΔH=−14.5 ± 0.15 kcal mol^{-1}, $T\Delta S$= −32.1 kJ mol^{-1} MukE monomers bound to a middle region of MukF have interacting surfaces with both monomers of a MukB head (total buried surface area estimated from *H. ducreyi* engaged heads complex (24) is 344 $Å^2$ and 630 $Å^2$)

Supplementary figure S4

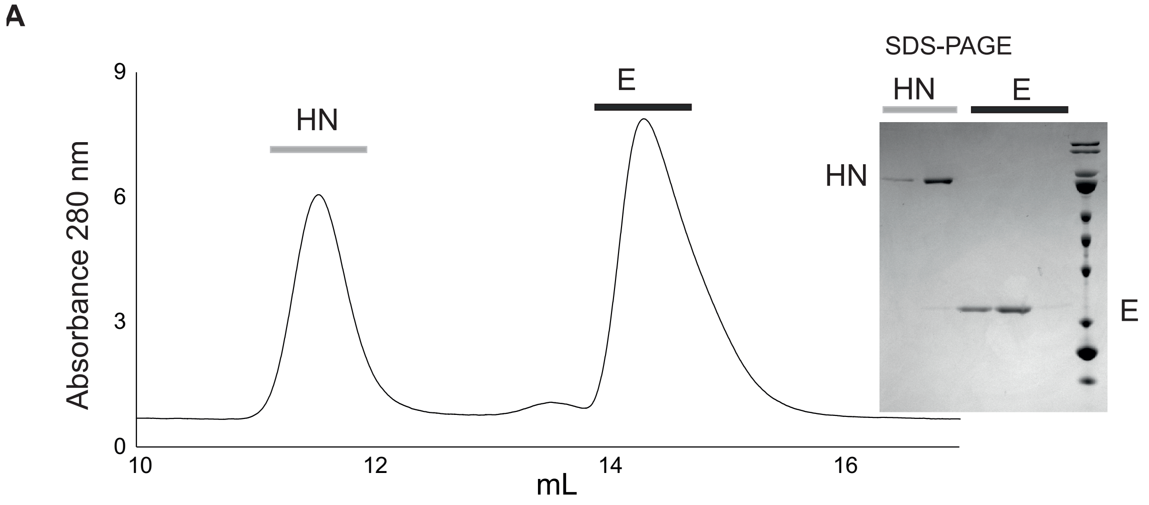

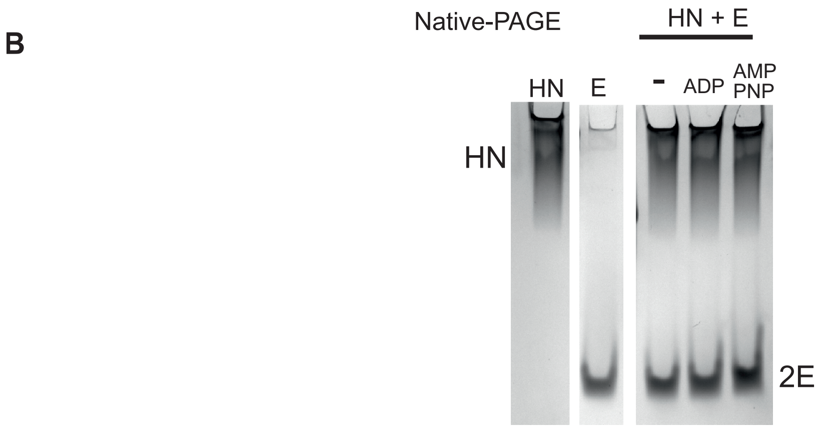

MukE requires MukF for stable interaction with MukB**. A** SEC of samples containing HN (10 µM) and MukE (10 µM). Inset, SDS PAGE of peak fractions as indicated. **B** Native PAGE of samples containing HN and MukE, in the absence and presence of the indicated nucleotides.

Supplementary figure S5

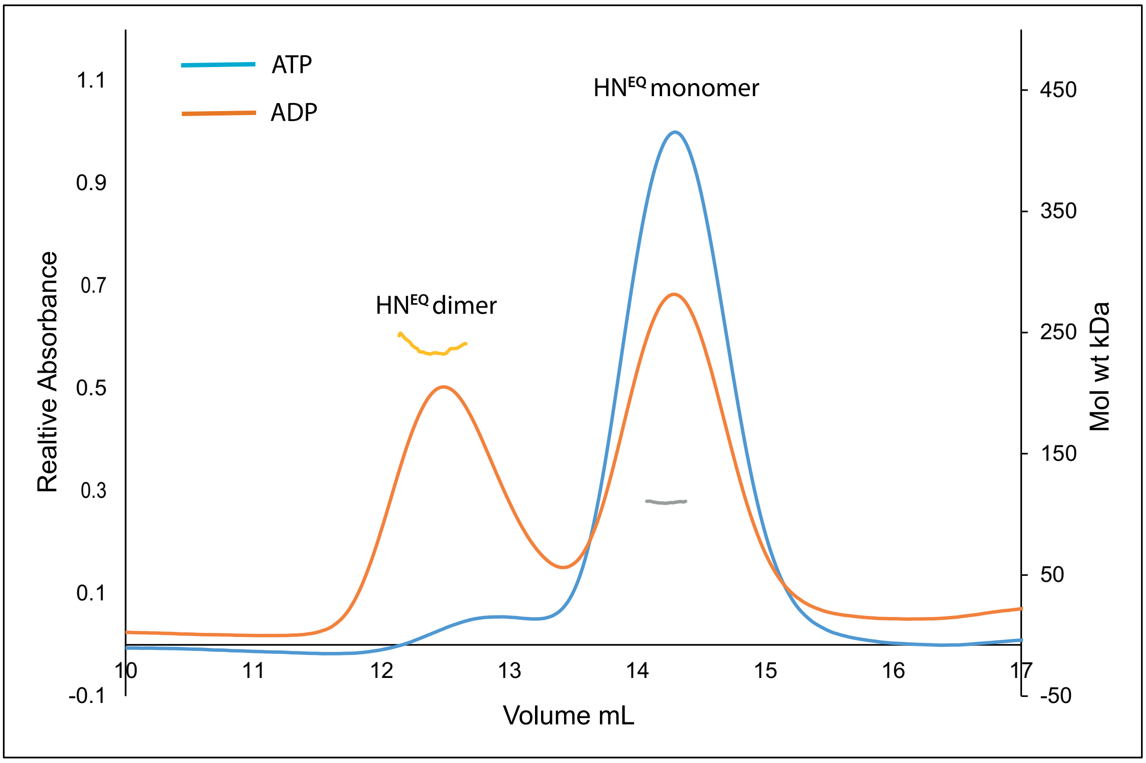

HN^{EQ} dimerises in the presence of ATP independently of MukF presence. The SEC-MALS analysis of samples incubated in the presence of ATP (orange trace) or ADP (blue trace) for 3hrs prior to resolution on Superdex 200 column.

Supplementary figure S6

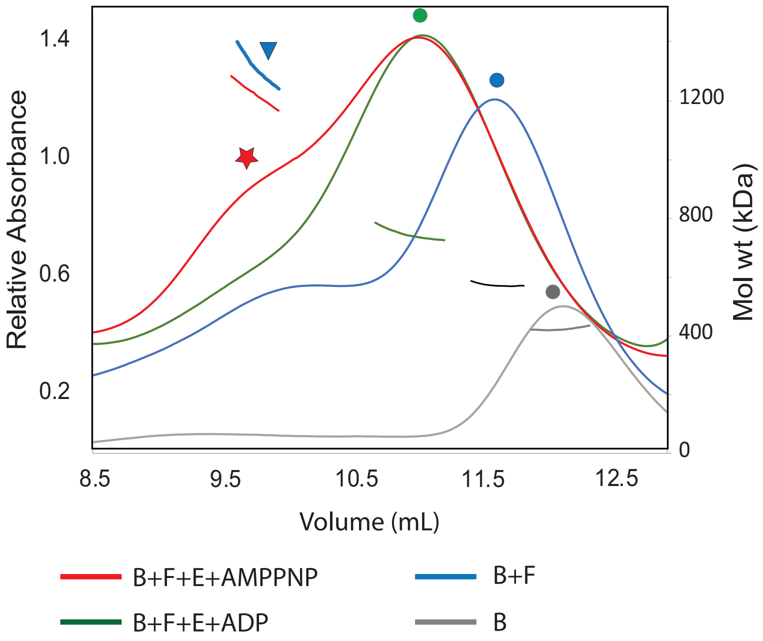

	Complex	Observed (kDa)	Predicted (kDa)
★	4B-2F-4E	1205 ± 35	951
●	2B-2F-4E	720 ± 21	587
▼	4B-2F/2B-4F	1252 ± 38	797/810
●	2B-2F	555 ± 16	469
●	2B	405 ± 28	364

MukBEF complexes generated with full length MukB can form dimer of dimers in the presence of AMPPNP. SEC-MALS of MukB, MukF and MukE complexes. The proteins were at concentrations 5µM B, 2.5µM F and 5µM E. Separation was through Superose 6 column. The observed masses are higher than those predicted. In the sample of B+F, this is partly because of likely higher order complexes containing additional F dimers, in addition to 4B-2F complexes (see main text).

Supplementary figure S7

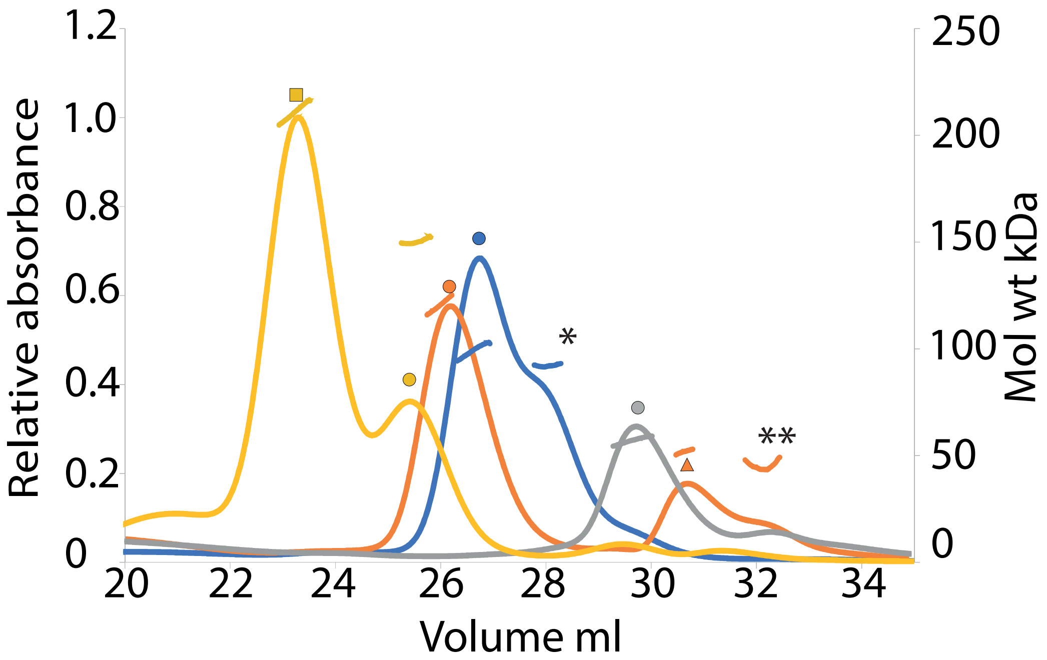

		Complex	Observed(kDa)	Predicted (kDa)
● (blue)	MukFWT	2F	99.2	105.4
● (yellow)	MukFWT&MukE	2F-2E	150.0	162.4
■ (yellow)		2F-4E	210.0	223.0
● (grey)	MukFm	1F	57.3	52.7
● (orange)	MukFm&MukE	1F-2E	120.0	140.9
▲ (orange)	MukE	2E	42.0	58.8

Monomeric MukF variant forms complexes with MukE *in vitro*. The samples containing 20 µM (monomer conc.) of MukF or MukF^{M} and 40µM MukE were separated on S200 column. A shoulder of a peak containing MukF (blue trace) reflects a presence of proteolytically degraded protein in this purified fraction (mass 93.4 kDa). The observed mass of MukE defers substantially from the predicted mass, most likely because the peak contains MukE in dynamic equilibrium of constantly exchanging monomeric and dimeric forms, as observed by others previously (31,32). A small shoulder off this peak (mass 32kDa) might be a monomeric MukE or a contaminant.

Supplementary figure S8

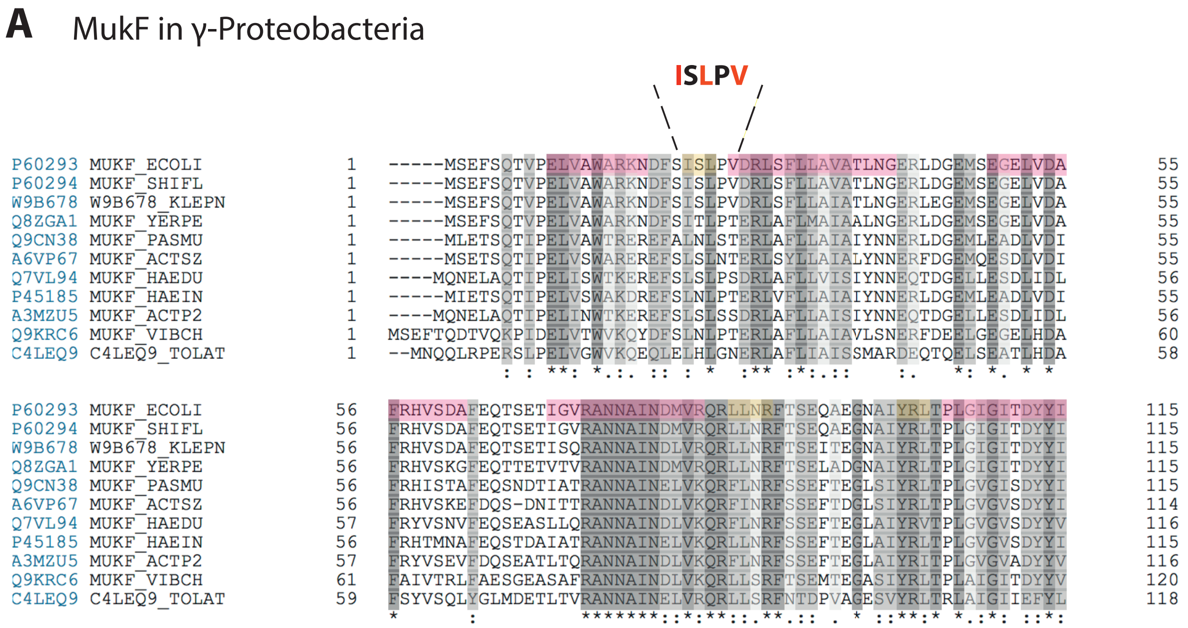

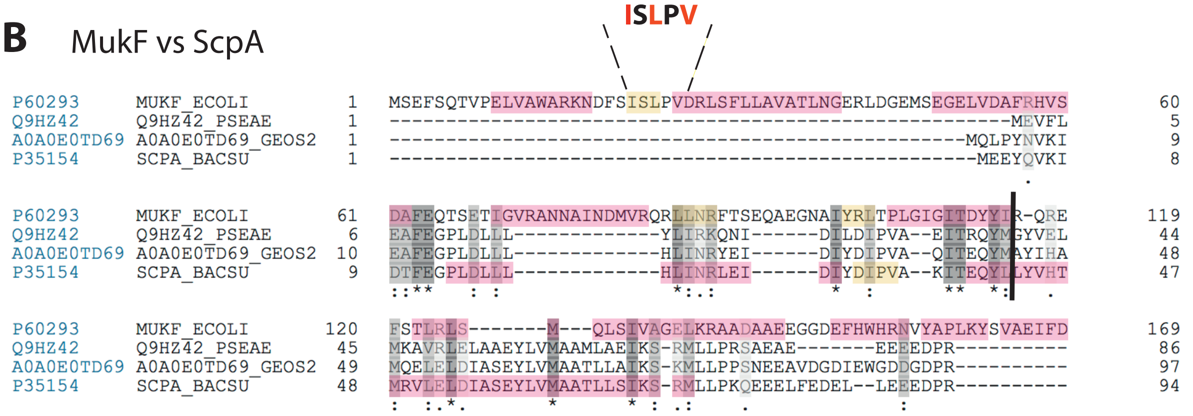

The structural elements supporting MukF dimerization interface are highly conserved in γ-Proteobacteria but absent in distant bacterial species. **A** An amino acid sequence alignment of MukF proteins from γ-Proteobacteria. Only the globular part of the N-terminal domain is shown. Conservation is highlighted in grey – the colour intensity parallels the level of conservation, with the highest level being indicated in darkest grey. The structural elements of *Ecoli* MukF are marked: α helics in pink - β strands in yellow. The sequence motif in dimerization interface is enhanced and mutagenized residues in $MukF^{M}$ are shown in red. **B**

The elements supporting MukF dimerisation domain are missing in kleisins from more distant bacterial species, such as *Pseudomonas aeruginosa*, *Geobacillus* sp. and *Bacillus subtilis*. The black bar marks the end of *E.coli* MukF globular N-terminal domain.

Supplementary table S1

Predicted mass of MukBEF complex components and their variants.

Protein variant	Predicted mass* (Da)	Predicted mass +ACP** (Da)
2MukB (-His tag)	345561.74	363521.45
HN WT(-His tag)<br>HN SR (-His tag)<br>HN EQ (-His tag)<br>HN ^{C*} (-His tag)	91584.7<br>91653.81<br>91583.72<br>91373.44	100564.57<br>100633.68<br>100563.59<br>100353.31
2F (-His tag)	105800.78	
2E (-His tag)	58734.06	
F^{M} (-His tag)	52891.25	
2FN10 (-His tag)	85515.48	

*The mass includes first methionine.

**MW ACP-4'phosphopanetheine 8979.87 Da

Supplementary table S2

Mass of MukBHN/MukB complexes with MukFE in presence of AMPPNP in mass spectroscopy assays.

Complex	Predicted mass (Da)	Observed mass (Da)*	Predicted mass with bound ACP-4'Ppant (Da)
2HN-2F-4E	406438	424831±45	424398
3HN-2F-4E	498023	526362±7	525468
4HN-2F-4E	589607	624470±47	626538

*The observed values represent masses of complexes determined in the sample illustrated in Figure 2E bottom panel. The mass of 2HN-2F-4E complex in samples without nucleotide or with ADP/ATP were 425555± 50, 425234± 45, and 424704±1Da, respectively. Predicted masses include ACP with its prosthetic group 4'phosphopantetheine (Angellini et al.2012) and AMPPNP (506Da) where appropriate.

Supplementary table S3

Mutated variants of MukB and MukF

**mutated variant**	**location in MukB/F**	**alteration(s) in MukB/F amino acid sequence**	**function impaired**
HN- SR	signature loop	S1366R	head engagement*
HN - EQ	Walker B	E1407Q	ATP hydrolysis**
HN^{C*}	cap	F1453S H1458A R1465A	Interaction with MukF C-ter domain***
HN^{N*}	neck	L1219K L1226K	Interaction with MukF 4HB**
F^{M}	dimerisation interface	I22T L24N V26T	MukF dimerisation***

* Woo et al, 2009, Badrinarayanan et al., 2012

** Zawadzka et al., 2018
*** this work

Supplementary table S4

MukB ATPase activity stimulated by MukF^{M}.

Protein	ATP molecules hydrolysed/ MukB dimer/min ± SD	% activity
MukF WT	23.3 ± 1.50	100
MukF^{M}	20.9 ± 0.89	90

Concentrations in assays were: MukB, 0.5µM, MukF/F^{M}1.25µM. The values represent averages of initial rates from 3 experiments ±SD.
